# A descriptive phenomenological study of school-related gender-based violence: lived experiences of symbolic violence, harassment, and systemic complicity in a mixed secondary school in Nairobi, Kenya

**DOI:** 10.1186/s12889-025-25341-0

**Published:** 2025-11-12

**Authors:** Aggrey Gisiora Mokaya, Gideon Kikuvi, Joseph Mutai, Peter Memiah

**Affiliations:** 1 Africa Health Research Institute, Nelson R. Mandela School of Medicine, 3rd Floor, K-RITH Tower Building, 719 Umbilo Rd, Umbilo, Durban, KwaZulu-Natal 4001 South Africa; 2 Centre for Public Health Research, Kenya Medical Research Institute, Nairobi, Kenya; 3 School of Public Health, Jomo Kenyatta University of Agriculture and Technology, Juja, Kenya; 4 University of Maryland School of Graduate Studies, Baltimore, MD USA

**Keywords:** School-related gender-based violence, SRGBV, Symbolic violence, Hierarchical power, Peer complicity, Qualitative study, Kenya, Adolescents, Custodial neglect, Psychosocial harm

## Abstract

**Background:**

School-related gender-based violence (SRGBV) affects students’ mental health, academic performance, and overall well-being. This study explored the lived experiences of adolescents facing SRGBV in a mixed secondary school in Nairobi County, examining the mechanisms through which gendered violence is perceived to be perpetuated and normalized.

**Methodology:**

A qualitative descriptive phenomenological approach was employed. Data were collected through two separate focus group discussions (one with six girls, one with six boys), comprising twelve Form 1 students aged 14–17 years (median = 15), who had joined secondary school two months before data collection. Discussions were audio-recorded, transcribed, and thematically analyzed using Braun and Clarke’s six-phase framework, with manual coding to ensure iterative engagement with the data.

**Findings:**

Participants perceived a pervasive culture of power imbalances and SRGBV within the school. They described older male students as physically excluding and verbally silencing younger peers, and coercive sexual behaviors as becoming normalized. Custodial figures, including teachers and parents, were described as engaging in survivor-blaming, fostering a culture of silence that reinforced gender hierarchies. Peer dynamics also shaped experiences, with some older female students reinforcing patriarchal norms and pressuring younger girls to conform. These interactions were perceived to contribute to psychosocial harm, including fear, isolation, mistrust of authority, and emotional withdrawal.

**Conclusion:**

In this school, participants described SRGBV as operating through interwoven structural and cultural mechanisms, with symbolic violence central to the internalization of harm and reproduction of inequalities. While these insights may resonate with wider patterns, they remain context-specific and exploratory. Addressing SRGBV requires interventions targeting custodial and peer complicity and the cultural normalization of violence. A holistic approach that combines structural reform, cultural transformation, and psychological care is critical to fostering equitable educational spaces.

**Supplementary Information:**

The online version contains supplementary material available at 10.1186/s12889-025-25341-0.

## Introduction

School-related gender-based violence (SRGBV) poses a significant threat to adolescents’ mental health, well-being, safety, and academic performance in secondary schools [1, 2]. SRGBV encompasses acts or threats of physical, psychological, or sexual violence rooted in broader gender norms and societal inequalities, occurring in and around schools and affecting access to and experience of education [3, 4]. While corporal punishment and bullying – including verbal, social, and physical victimization – have long been central to discussions of school violence [5–7], SRGBV reflects a deeper – often covert – entanglement of gendered power dynamics that are either newly manifesting and recognized in school contexts or increasingly documented in research and policy [8].

Globally, SRGBV spans diverse cultural, economic, and geographic contexts, highlighting its universality and the systemic nature of its underlying causes [9–11]. UNESCO estimates that approximately 115 million children and adolescents experience SRGBV, with girls disproportionately affected [12, 13]. In Africa, SRGBV is widespread and disproportionately affects girls, exacerbating gender inequalities and limiting educational attainment [14–17]. In Ethiopia it has been reported that the prevalence of SRGBV is as high as 47.2% [18]. In Kenya, the problem is particularly notable in Nairobi County, where entrenched gender norms and societal expectations perpetuate gender-based violence. A study in Kenya showed that nearly 40% of female students frequently experience harassment from both peers and teachers, fostering a culture of fear and silence [19].

The consequences of SRGBV extend beyond the immediate experience of violence, affecting adolescents’ psychological, emotional, physical, and social well-being [17, 20]. In the short term, many survivors report fear, shame, and social withdrawal, as well as physical injuries or somatic symptoms such as headaches and sleep disturbances [21]. Social consequences include disrupted peer relationships and isolation. Over the medium to long term, SRGBV has been linked to chronic anxiety, depression, and post-traumatic stress disorder (PTSD), with cumulative effects that manifest as disengagement in the classroom, declining academic performance [20], and, in some cases, school dropout [22].

Emotionally, many survivors experience fear, shame, and social withdrawal, which limit their ability to engage socially or seek help [1, 15]. Socially, SRGBV disrupts peer relationships, fosters isolation, and reinforces harmful gender norms that hinder personal development and academic success. These cumulative effects often manifest as disengagement in the classroom, declining academic performance, and, in some cases, school dropout. Adolescents subjected to SRGBV often avoid school or specific areas within the school due to fear, further exacerbating their educational struggles [23]. The stigma associated with gender-based violence deters survivors from seeking support, increasing their isolation and vulnerability. SRGBV also perpetuates social inequities, particularly for marginalized groups, by limiting access to education and opportunities for upward mobility [24].

In many contexts, including Kenya, the intersection between SRGBV and general bullying complicates efforts to address school violence, as the local context of routine bullying victimization likely obscures the gendered dimensions of SRGBV. This is especially, in Kenya, true given the high prevalence of routine bullying victimization at over 80% [25, 26]. This is further reinforced by the normalization of gender-based violence in the wider society, particularly in low-income areas, where GBV rates remain significantly high (20.5%) [24] and are often perceived as part of everyday life [24, 27]. Consequently, what is recognised as “violence” within schools is often ambiguous [28]. Most policy and intervention frameworks align with the majority perception that school violence is primarily physical or verbal victimization, meaning that gendered forms such as SRGBV are frequently excluded from the scope of what is considered [25]. Existing anti-bullying measures, therefore, largely focus on managing physical confrontations and verbal aggression, while failing to account for the subtler, yet equally harmful, gendered dimensions of violence [28]. This oversight allows SRGBV to remain hidden and insufficiently addressed within school environments. frequently reported being directly affected through sexual harassment and social exclusion [29], while boys described being affected indirectly through pressure to conform to aggressive masculine norms, which reinforced harmful gender dynamics. The failure to recognize and tackle these gendered dimensions of violence perpetuates a cycle where SRGBV continues to be normalized and embedded within the school culture.

This study sought to explore how adolescents in a co-educational secondary school in Nairobi County experienced SRGBV, focusing on the gendered mechanisms of violence. By analyzing the lived experiences of students affected by SRGBV, the study aimed to uncover the social and cultural factors that perpetuate gendered violence within schools and how these experiences influenced students’ psychosocial well-being.

## Methodology

### Study context

This study was conducted in a mixed-sex (co-educational) public secondary school situated near a low-income informal settlement in Nairobi County, Kenya, in March 2020, just before the nationwide COVID-19-related school closures. The school served a socioeconomically vulnerable population, making it an important site for understanding how bullying victimization unfolds in daily school life. Participants were Form 1 students who had joined the school in January of the same year. New entrants were purposively selected because prior research indicates that Form 1 students are especially vulnerable to bullying and harassment during their first term, a type of hazing termed in Kenyan slang (*Sheng*) as *monolization* [30, 31]. The study was nested within a larger project evaluating the effects of a teacher-led psychoeducation intervention on bullying victimization [25], depression [32], and suicidality [33, 34], using a two-group post-test only design. In the intervention schools, psychoeducation sessions were delivered to all students in Forms 2 to 4 before Form 1 students arrived, recognizing that older students are often the most frequent perpetrators of school violence, and that the most severe bullying in Kenyan secondary schools typically targets Form 1 students during their first term. This substudy was conducted in a control school where the intervention was not implemented, representing the unaltered school environment or “situation as usual.” While the original objective of the qualitative component was to explore bullying victimization broadly, the theme of school-related gender-based violence (SRGBV) emerged inductively during data collection and analysis.

### Study design

A descriptive phenomenological design was adopted to explore the lived experiences of adolescents related to SRGBV. The phenomenological approach was chosen because it focuses on understanding participants’ subjective experiences and the meanings they attach to those experiences [35]. Given that SRGBV is rooted in both personal experiences and collective cultural contexts, this design allowed for in-depth exploration of individual perceptions while acknowledging shared patterns within the group. The design is well-suited for studies that aim to uncover how individuals experience and make sense of violence, power, and gender dynamics in a school setting [35, 36].

### Participants

The participants were 12 adolescents (6 girls and 6 boys), purposively selected from the Form 1 cohort who had entered secondary school in January 2020. Purposive sampling was used to ensure gender balance and to capture the perspectives of students most at risk of bullying and harassment during their transition to secondary school. A summary of participant characteristics is shown in Table 1.

**Table 1:** Sociodemographic characteristics of study participants

Participant	Pseudonym^*^	Age group (in years)	Gender
B1	Odhiambo	16–17	Male
G1	Achieng	14–15	Female
B2	Mwangi	14–15	Male
G2	Wanjiku	16–17	Female
G3	Kerubo	14–15	Female
G4	Naliaka	14–15	Female
B3	Kiprotich	14–15	Male
B4	Baraka	16–17	Male
G5	Naserian	16–17	Female
G6	Amina	14–15	Female
B5	Were	14–15	Male
B6	Abdi	14–15	Male
Summary		Median (15 years)	Male (50.0%); Female (50.0%)

^*^Not their real names

### Data collection

Focus group discussions (FGDs) were used to explore adolescents’ experiences of bullying victimization and peer dynamics. While SRGBV was not the initial focus, it emerged during analysis as a critical and recurring theme. Two FGDs were conducted in March 2020, one with boys and one with girls, each lasting approximately 90 min. The groups were separated by gender to create safer and more comfortable environments for open expression. The sessions were run concurrently in separate classrooms to minimize disruption to learning. Both were held in private, familiar school settings and facilitated by gender-matched moderators to promote open discussion (a male moderator for the boys’ group and a female moderator for the girls’ group). The FGDs were scheduled during regular school hours, which also made individual interviews infeasible given time constraints, as per the authorization granted by the Ministry of Education. Short time is often a constraint when collecting data in educational settings [37, 38].

FGDs were selected for their ability to elicit communal experiences, particularly relevant to understanding peer-based phenomena like bullying and SRGBV, which unfold in shared school spaces. Though phenomenological research is often associated with individual interviews, scholars argue that focus groups can meaningfully surface lived experience when the phenomenon is social in nature [39–41]. In the Kenyan context, FGDs have also been shown to support disclosure of sensitive issues among adolescents [42], especially when stratified by gender and led by relatable facilitators [43]. While in-depth individual interviews might have allowed for deeper exploration of traumatic experiences, FGDs were judged to be the most appropriate method in this school setting. Bullying and SRGBV often occur in collective spaces such as classrooms, corridors, and dormitories, making them particularly suited to group-level discussion. Moreover, the practical realities of the school timetable and Ministry of Education restrictions limited opportunities for extended one-on-one interviewing. By conducting single-gender FGDs with gender-matched facilitators and in the presence of the school counselors, we sought to balance the sensitivity of the topics with the need to capture shared perceptions, social norms, and group dynamics that shape bullying and SRGBV, by extension.

All FGDs were audio-recorded with participants’ consent and transcribed verbatim. The discussions were conducted in a mix of Swahili and English, with frequent use of Sheng (local slang); these language shifts were preserved in transcription and translated during analysis to retain cultural meaning. To protect confidentiality, identifying information was removed and pseudonyms were assigned. The group setting enabled participants to co-construct meaning around school-based violence, illustrating how SRGBV is experienced and interpreted within their shared social context and lived experience. Given that only two FGDs were feasible within this study context, we do not claim theoretical saturation. Instead, the findings should be understood as exploratory, hypothesis-generating insights into how SRGBV was perceived by students in this control school.

### Data analysis

Data were analyzed using Braun and Clarke’s six-phase framework for thematic analysis [35, 44], which allowed for systematic identification of themes related to SRGBV, gendered power dynamics, and peer complicity. This study employed manual coding using Microsoft Word. Manual coding was justified by the small sample size and the depth of the data, which allowed researchers to achieve meaningful analysis. The manual coding process involved reading and highlighting sections of the transcripts directly within Microsoft Word documents, where color-coded highlights and margin comments were used to organize initial codes. While software-based approaches offer benefits such as streamlined retrieval of codes and themes, the manual process facilitated deeper engagement with the text and flexibility in adjusting codes and categories during the analysis process. Throughout this paper we use the term “survivor” rather than “victim,” to avoid framing participants as passive or lacking agency. While participants themselves did not use either term explicitly, we interpret their accounts through a survivor-centered lens, consistent with qualitative approaches that prioritize dignity and resilience.

Familiarization with the data involved multiple readings of the transcripts, allowing researchers to identify initial impressions and patterns related to power assertion, coercion, and survivor-blaming. This immersion laid the groundwork for manual, line-by-line coding in Word, ensuring a detailed engagement with the data. Codes captured both explicit expressions of violence and subtle mechanisms of control. Some of the examples from the codes include: “fear-driven silencing” emerged from statements said to Naserian one of the participants a perpetrator *“Try doing that*,* and I will slap you”* (female, 16–17 years), while “custodial neglect and survivor-blaming” were exemplified by quotes such as *“The teacher told me to dress properly if I didn’t want attention”* (Kerubo, female, 14–15 years). “Coercive expectations” reflected instances where harassment was trivialized, as illustrated by *“Sometimes they purposely touch your breasts or rear*,* and then they say sorry*,* it was an accident”* (Naliaka, female, 14–15 years).

In the next phase, codes were grouped into themes based on shared meanings. Intimidation, silencing, and power dynamics were clustered under Power Assertion and Hierarchical Violence, while custodial neglect and inaction formed the theme Systemic Complicity and Suppression. The reviewing stage involved refining themes for coherence and distinctiveness, distinguishing, for instance, peer-driven harassment gatekeeping from institutional neglect by custodial figures. The final themes – Power Assertion and Hierarchical Violence, Sexual Harassment and Normalization of Coercion, Systemic Complicity and Suppression, Reinforcement of Violence, and Psychosocial and Internalized Harm – were defined to reflect the structural, cultural, and psychological dimensions of SRGBV experiences among these learners (Supplementary File).

**Fig. 1:**
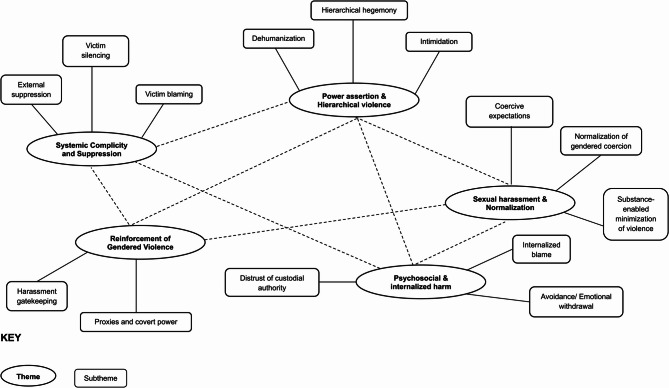
Thematic map showing mechanisms of SRGBV in the study

It is important to note that Braune and Clarke’s approach of reflexive thematic analysis not only describes semantic content but also interprets latent meanings and underlying mechanisms in the data. This means moving beyond stating that harassment occurs to elucidating how it operates through coercion, peer shaming, or suppression [44]. In line with other studies of school violence and SRGBV, we sought to contextualise participants’ narratives within broader structural and cultural dynamics. This analytic step is essential to avoid presenting violence as isolated incidents and instead to explain how systemic mechanisms reproduce harm. Thematic categories were illustrated with direct quotes and synthesized into a thematic map, visually demonstrating how hierarchical power structures, peer complicity, and custodial neglect interact to perpetuate long-term harm (Fig. 1).

## Findings

The findings of this study reflect adolescents’ perceptions and lived experiences of school-related gender-based violence (SRGBV) within a co-educational secondary school in Nairobi County. Based on participants’ narratives, five interconnected themes emerged through thematic analysis. These themes should be understood as subjective accounts rooted in participants’ lived experiences, rather than as objective or generalizable findings, capturing how SRGBV was understood and experienced at structural, interpersonal, and psychological levels. It is important to note that girls provided more direct and detailed accounts of SRGBV, whereas boys’ contributions more often reflected bystander perspectives or broader bullying dynamics. This imbalance itself is telling, highlighting how experiences of SRGBV were gendered in their narration. According to the students in this school, violence was deeply embedded in the school environment, reinforced by hierarchical power dynamics, normalized coercion, and perceived institutional neglect. The thematic map, grounded in these accounts, illustrates the interplay between power, peer relations, and systemic inaction in sustaining SRGBV. It is suggestive of a need for interventions that respond to both individual experiences and the broader school structures within which these harms were seen to occur (Fig. 1). Please note that the names used are pseudonyms and not the real names of the participants.

### Power assertion and hierarchical violence

This theme captures the structural mechanisms of SRGBV, where hierarchical power dynamics within this school’s environment allowed senior students, primarily boys, to dominate and control younger students through both physical and verbal means. The violence was not random but embedded in the school’s social fabric, perpetuated through exclusion, intimidation, and fear-driven compliance.

One participant described their experience of how older boys controlled access to communal resources through physical dominance:

*“When going for lunch*,* those big boys from Form Three and Form Four come and push you back*,* and you can’t do anything…”* (Naliaka, female, 14–15 years).

Another boy detailed how the threat of violence extended into daily routines such as mealtime, where food was seized and intimidation normalized:

*“Unaona lunch time*,* they… wanakutumia. Sasa tuseme hakuna maprefects wengi pahali*,* wanachukua chakula yako*,* wanagawana. Unabakishiwa kama two spoons*,* na unaambiwa ukijaribu kusema*,* utaona.” (You see*,* at lunchtime they gang up on you. If there aren’t many prefects around*,* they take your food and share it among themselves. You’re left with just two spoonfuls and warned that if you say anything*,* there’ll be consequences*.) (Kiprotich, male, 14–15 years).

This exclusionary practice reflected the systematic nature of hierarchical violence, where younger students, especially girls, were subordinated in public spaces and denied the agency to resist. Verbal domination was another form of control, as seen in the use of infantilizing language to silence and demean younger students:

*“…Say someone in the field hits you with a ball*,* he says wee mtoto nyamaza (Shut up*,* you child).”* (Odhiambo, male, 16–17 years).

Fear was a critical mechanism by which perpetrators ensured compliance. Attempts by survivors to report harassment were often met with retaliation:

*“Sometimes you are walking down the stairs*,* and then a boy kicks you. When you tell him*,* ‘I will report to the teacher that you caused me to fall*,*’ he tells you*,* ‘Try doing that*,* and I will slap you….’”* (Naserian, female, 16–17 years).

The strategic use of fear extended beyond immediate encounters, with premeditated violence maintaining long-term control over survivors:

*“…They will wait for you outside the school*,* or they start planning and getting information. You start fearing even in school or wanakupiga (they beat you up).”* (Naserian, female, 16–17 years).

Here Wanjiku interjects to add that:

..*Or anatuma watu wenye anajua wanakuanga wabaya… (He sends his dangerous associates who waylay you.) He won’t be there so you won’t know it’s him. Then ukimsuspect (if you suspect him) he starts saying he wasn’t uiliniona (Did you see me there)? I wasn’t there* (Wanjiku, female, 16–17 years).

These examples demonstrate how hierarchical violence was systematically maintained, creating an environment where survivors were silenced and perpetrators acted with impunity.

### Sexual harassment and normalization of coercion

This theme highlights how sexual harassment became embedded in everyday school interactions through coercive social expectations and the normalization of gender-based violence. Coercive social expectations refer to the subtle but powerful pressures that dictate how individuals, particularly girls, should respond to harassment. These expectations frame unwanted advances as routine social exchanges, positioning resistance as an overreaction or an act of defiance. Survivors who rejected advances risked social consequences, such as ridicule or exclusion, making it difficult to assert personal boundaries. This environment fostered a culture where enduring or dismissing harassment is seen as the expected response, while refusal invites further victimization. Perpetrators often leveraged these social norms to justify their behaviour, framing inappropriate contact or comments as harmless, accidental, or even affectionate. By doing so, they shifted responsibility away from themselves and invalidated the distress of those they targeted. In some cases, survivors found that rejecting advances led to verbal humiliation, further discouraging them from speaking out or resisting. The social climate reinforced these patterns, making harassment appear like an unavoidable aspect of school life rather than a violation of personal autonomy, including harassment behaviour being attributed to substance use.

One participant described how unwanted physical contact was routinely dismissed:

*“Sometimes they purposely touch your breasts or rear*,* and then they say sorry*,* it was an accident. Can an accident happen every day?”* (Naliaka, female, 14–15 years).

By minimizing the severity of their actions, perpetrators deflected responsibility and invalidated survivors’ distress. Additionally, another participant pointed to how reported substance use among older boys further enabled the normalization of such harassment, including implied assertion that boys are expected to cope with some level of harassment:

“*Na some of these guys… tuseme hii shule kuna wale ambao wana abuse drugs. Unajua saa ukiabuse drugs*,* mental yako venye unakaa. Kuna ninii apo*,* challenges*,* so unajua mtu kama huyo haskii vibaya na haoni kitu kubwa – yaani big deal – kushika msichana. Unajua wasichana ndo wakona iyo shida. Kijana unaeza manage kama sisi vijana wadogo. Huyu msichana ni msichana*,* hana nguvu*,* yaani tu sijui nisemeaje. (Some of these guys… let’s say in this school there are some who abuse drugs. And you know when someone abuses drugs*,* their mental state changes. They face certain challenges*,* so someone like that doesn’t feel bad or even see it as a big deal to touch a girl. You see*,* it is the girls who really face that problem. A boy might be able to handle it*,* like we smaller boys. But a girl is a girl*,* she is powerless*,* I don’t even know how to explain it.)”* (Mwangi, male, 14–15 years).

Verbal degradation accompanied rejection of advances, serving as a coercive mechanism:

*“…Sometimes you are walking within the school*,* and boys in Form 3 and 4 will corner you somewhere and want to touch you*,* to touch your breasts or your buttocks. If you refuse*,* they start saying*,* ‘Ona huyu sura mbaya (Look at this ugly one).’ It makes me feel bad and sometimes I just sit in class and think why God let my parents bring me to this school.”* (Naliaka, female, 14–15 years).

The use of peer-shaming as a form of coercion pressured survivors into compliance to avoid public humiliation. This point was echoed and elaborated by other girls in the group, showing how FGDs allowed participants to build on each other’s accounts. For example, after Naliaka’s statement above, Naserian added her own experience:*“…Like the other day*,* I was leaving school*,* I took my bag*,* and was heading downstairs when a boy came up to me and said*,* ‘Why don’t you give me a hug?’ I told him*,* ‘I don’t like that behaviour.’ Then he started saying*,* ‘Oh*,* a hug is like a greeting for us….’”* (Naserian, female, 16–17 years).

This exchange highlights how multiple participants reinforced one another’s accounts, illustrating that unwanted advances were often normalized and framed as harmless social interactions. While girls narrated these incidents as direct personal experiences, boys in their group spoke more often as bystanders. For example, one boy explained that substance use among older students made harassment seem trivialized, but framed this primarily as something that affected girls rather than himself. This contrast shows how girls provided first-hand accounts of harassment, whereas boys largely described the problem as something happening around them. This difference in positioning highlights the gendered nature of SRGBV narratives in the FGDs.

### Systemic complicity and suppression

Systemic complicity refers to the role of custodial figures, including teachers and parents, in perpetuating SRGBV through survivor-blaming and the suppression of reporting. As narrated by the participants, rather than providing support, these it was often felt that these figures shifted blame onto survivors, reinforcing the legitimacy of violence.

One participant recounted how a teacher’s response to harassment perpetuated harmful gender norms:

*“…The teacher told me to dress properly if I didn’t want attention.”* (Kerubo, female, 14–15 years).

This form of survivor-blaming by authority figures deflected responsibility away from the perpetrator and placed the burden on the survivor to prevent harassment. Similarly, family-based survivor-blaming further silenced survivors:

*“Nilijaribu kuambia my mum that hawa maboys wamekuwa wakinishikashika. Alinislap na akaniambia*,* ‘Si nilikuambia uwache hii umalaya uko nayo!*’ *(…I tried telling my mum that these boys were touching me. She slapped me and said*,* ‘Didn’t I tell you to stop being promiscuous?’”* (Kerubo, female, 14–15 years).

Kerubo explained that she had initially approached her mother hoping for support, but instead the conversation turned into moral judgment. This experience discouraged her from ever seeking help again, leaving her feeling ashamed and isolated. Such custodial victim-blaming illustrates how parental rejection can reinforce internalized blame and silence survivor.

Similar themes also emerged with the boys:

*“I don’t talk to teachers anymore because they don’t help…”* (Baraka, male, 16–17 years).

Odhiambo (male, 16–17 years) interjected and added to Baraka’s sentiment saying:

“Uhmmm…. Some teachers ignore you when you report bullying to them. They tell you it’s not a big thing just deal with it as a man since some of these challenges you will face anywhere.”

Baraka added further that;

*“…There are students who*,* when they see you talking to teachers*,* think you are ‘snitches.’ But teachers also can’t be trusted for they tell other teachers issues to do with students and this makes some students go mute on reporting bullying in the school.” (Baraka*,* male*,* 16–17 years)*.

These responses reflect custodial neglect, where both school authorities and parents failed to provide protection and instead perpetuated harm. The long-term influence of this neglect was evident in the erosion of trust. Fear of retaliation further limited survivors’ ability to report incidents or hold perpetrators accountable:

*“If you are appointed as a prefect and you write their names down as noisemakers and take them to the teacher*,* they will wait for you outside the school… [to deal with you].”* (Naserian, female, 16–17 years).

Achieng shared a further example of how retaliation extended beyond school spaces:

“*Yes…you have to go with your sister or brother or someone who is older than you to protect [you] from those people. Let’s say we went to a CU [Christian Union meeting] and talked about something that hurt them something that they do they want to beat you up. Me what I do I look for somebody mwenye tunaishi nayeye (who is my neighbour) then we go home because I fear those girls*” (Achieng, female, 14–15 years).

This external suppression highlights how the threat of violence extended beyond the immediate school environment, undermining survivors’ safety and further silencing them.

### Reinforcement of violence

According to participants, peer complicity played a crucial role in sustaining SRGBV through the reinforcement of patriarchal norms and harassment gatekeeping. In their narratives, younger girls described being encouraged by older girls and male peers to accept harassment. This encouragement, as perceived by participants, took the form of ridicule, shaming, and subtle coercion, cultivating a social environment in which resignation to harassment was normalized, and resistance was framed as deviant or naive. Participants recounted that some older girls framed such experiences as routine and unavoidable aspects of school life. Rather than identifying these encounters as forms of violence or challenging them as unacceptable, they often positioned submission as a marker of maturity. In doing so, they not only legitimized male dominance but also contributed to the intergenerational transmission of these harmful practices within the school setting.

One participant described how older girls ridiculed younger girls for resisting harassment:

*“Some of the older girls just accept to be touched and they laugh at us and say*,* ‘Nyinyi ni washamba*,* kuja tuwafunze (You are backward*,* come*,* let us teach you).’ Hadi unapata msichana anakalia juu ya kijana hadi unafikiria…(You even find a girl sitting on a boy and you are left thinking…) ”* (Naliaka, female, 14–15 years).

Naserian added that.

*Eeh (Yes)… a boy anakaa hivi apart and then msichana anakalia (A boy sits like this with his legs apart and then the girl sits in between).*

This reflects younger students’ perceptions of how older peers positioned harassment as normal, rather than direct accounts from older students themselves. This perceived harassment gatekeeping served to maintain male dominance by socializing younger girls into resigning themselves to the situation. Covert forms of control, such as the use of threats through proxies, further reinforced violence:

*“Or anatuma watu wenye anajua wanakuanga wabaya… (He sends his dangerous associates who waylay you.) He won’t be there so you won’t know it’s him. Then ukimsuspect (if you suspect him) he starts saying he wasn’t uiliniona (Did you see me there)? I wasn’t there.”* (Wanjiku, female, 16–17 years).

The combination of peer reinforcement and covert intimidation created an environment where violence was continuously reproduced and normalized.

#### Psychosocial influence and internalized harm

Prolonged exposure to SRGBV had severe psychological and emotional consequences for survivors, leading to internalized blame, emotional withdrawal, and long-term mistrust of authority figures. One participant described how they coped with the distress caused by harassment:

*“…Some of the older girls just accept to be touched and they laugh at us and say*,* ‘Nyinyi ni washamba*,* kuja tuwafunze’ (You are backward*,* come*,* let us teach you). I ran away and hid myself in the toilet and I was thinking about what they were saying.”* (Naliaka, female, 14–15 years).

This highlights avoidance and emotional withdrawal as common coping mechanisms among survivors. Family-based survivor-blaming further exacerbated feelings of guilt and isolation. As also illustrated by Kerubo in the in the systemic complicity and suppression theme, parental reactions could reinforce silencing, compounding the effects of peer coercion.

Within the girls’ group, participants often shared feelings of shame or self-blame, while in the boys’ group, mistrust of teachers was more frequently emphasized. This suggests that while girls internalized harassment through avoidance and self-criticism, boys were more likely to articulate frustration at custodial figures or authority structures. Such contrasts between groups further demonstrate how gender shaped the interpretation of SRGBV experiences. The cumulative influence of these experiences led to long-term disengagement and mistrust:

*“I don’t talk to teachers anymore because they don’t help….But teachers also can’t be trusted for they tell other teachers issues to do with students and this makes some students go mute on reporting bullying in the school.”* (Baraka, male, 16–17 years).

This mistrust of authority figures reflects the broader failure of custodial structures to provide adequate protection and support in this school.

## Discussion

As narrated by participants, this study underscores the pervasive and complex nature of school-related gender-based violence (SRGBV) in a co-educational secondary school, illustrating how hierarchical power dynamics, peer complicity, and custodial neglect intersect to create ongoing harm. To better understand how SRGBV becomes embedded within the school environment, the findings are framed within Bourdieu’s theory of symbolic violence, which refers to the subtle, often unrecognized forms of domination and injustice that operate through social norms, cultural expectations, and internalized beliefs [45, 46]. Symbolic violence is particularly insidious because it does not rely on overt force; rather, it shapes perceptions and behaviours in ways that make inequalities appear natural and unquestioned [47, 48]. In the context of SRGBV, The data suggest that participants perceived harassment and abuse as inevitable within education. This reflects normalization rather than endorsement: participants described resignation to these practices, but not that they viewed them as acceptable [49]. By internalizing these power structures, individuals unknowingly sustain the cycle of violence, reinforcing gendered hierarchies and ensuring the transmission of SRGBV across generations [49]. Understanding SRGBV through this lens highlights the need to challenge not only explicit acts of violence but also the underlying cultural and institutional mechanisms that normalize and perpetuate it. In discussing these findings, we deliberately use the term “survivor.” This reflects both an ethical commitment to avoiding deficit language and a theoretical framing that recognizes participants as active agents negotiating symbolic violence, rather than passive “victims.”

The study underscores the role of custodial figures – teachers and parents – in perpetuating violence through survivor-blaming and neglect. Survivors who sought help from teachers were often met with dismissive or moralizing responses, reflecting the internalization of patriarchal norms by authority figures. Similarly, parents, instead of offering support, frequently engaged in moral policing. Such responses align with findings from global studies, such as those by Bhana (2015) in South Africa [50] and Leach et al. (2014) in Ghana [20], which demonstrate that survivor-blaming and custodial neglect are common across various socio-cultural contexts, possibly within the wider socio-cultural context in Kenya – where survivor-blaming and perpetration of gender-based violence by those who hold power have been demonstrated in literature [51, 52]. By transferring responsibility for violence onto survivors, custodial figures arguably create an environment where perpetrators feel emboldened to act without fear of reprisal.

This dynamic contributes to what Fraser (2000) terms institutional misrecognition [53], where institutions fail to acknowledge the lived realities of survivors and, in doing so, perpetuate harm. The absence of confidential reporting mechanisms and supportive interventions further compounds the issue, as survivors internalize the belief that their experiences are not worth reporting. This erosion of trust in custodial figures results in survivors withdrawing from potential sources of support, thereby inhibiting resistance or help-seeking and reinforcing long-term psychosocial harm [21].

Power assertion and hierarchical violence were central themes in this particular context, with older boys perceived to dominate shared spaces and using intimidation to control younger students. This mirrors findings from studies in sub-Saharan Africa, which reveal that school hierarchies often mirror societal power dynamics, privileging older males while marginalizing girls and younger students [1]. Through physical exclusion, verbal silencing, and threats of retaliation, older boys appeared to maintain their dominance and socialize younger boys into reproducing these behaviours when they ascend to senior positions [54, 55]. Hierarchical violence was also shown to operate through external suppression mechanisms, where threats extend beyond the school’s administrative boundaries. This dynamic discourages female students, particularly prefects, from assuming leadership roles due to fears of retaliation. This may be indicative of the broader societal norm that discourages female leadership and reinforces traditional gender roles. Addressing these issues requires interventions that challenge gender hierarchies both within and beyond school settings.

As described by younger participants, peer complicity played a significant role in perpetuating SRGBV. In their accounts, some older girls were perceived to act as gatekeepers of harassment by encouraging compliance among younger girls. While these are perceptions rather than first-hand accounts from older girls, they illustrate how younger students interpreted peer dynamics. Similar findings have been reported in studies by Parkes et al. (2023) and Ngidi and Moletsane (2023) [10, 17], which show that peer policing functions as a self-reinforcing mechanism within school environments. By aligning with male perpetrators, older girls reduce their own vulnerability to further targeting, thereby perpetuating the cycle of violence.

The gendered nature of peer policing is indicative of symbolic violence, as survivors of harassment themselves become agents of its reproduction. Bourdieu’s framework explains how individuals internalize their subjugation to the extent that they participate in enforcing it on others. This complicity ensures that patriarchal hierarchies remain intact and resistant to change. Peer education programs that challenge internalized gender norms and promote bystander intervention are critical to breaking this cycle [56]. The normalization of sexual harassment through social and peer mechanisms is another key finding of this study. Perpetrators often framed harassment as accidental or trivial, making it difficult for survivors to resist or seek help. This reflects what Bhattacharjee et al. (2020) describe as negative social norms – including around implementation of protective laws – leading to trivialization of violence, where gender-based harm is minimized or dismissed as benign [24]. Social coercion, particularly in the form of public shaming and verbal degradation, further pressures survivors to comply with unwanted advances.

Studies conducted in Kenya [57, 58] have highlighted similar dynamics, showing that harassment framed as socially normative interactions discourages reporting and fosters a culture of silence. Fraser’s concept of misrecognition may be instructive here [59], as survivors’ experiences are systematically denied or devalued within the school environment [45]. Addressing this requires shifting the cultural perception of harassment and coercion through gender-sensitive policies and educational interventions.

Prolonged exposure to SRGBV has severe psychosocial consequences, including emotional withdrawal, fear, and self-blame. Survivors often cope by avoiding authority figures and isolating themselves from their peers. This aligns with findings by Devries et al. (2014) and Parkes et al. (2023) [17, 21], who report that SRGBV is associated with increased risks of depression, anxiety, and social withdrawal. The internalization of harm reflects Bourdieu’s concept of symbolic violence, where survivors come to perceive their suffering as normal or inevitable, thereby limiting their ability to resist or seek intervention.

This study provides important insights into school-related gender-based violence (SRGBV), but several limitations should be noted. The study was part of a larger evaluation of a psychoeducation intervention delivered to students in Forms 2 to 4, targeting those most likely to bully new students. The focus group discussions (FGDs) reported here took place in a control school that did not receive the intervention. They reflect the usual conditions faced by new Form 1 students at the start of the school year. The study originally aimed to explore bullying victimization, and SRGBV emerged unexpectedly during data analysis. Planned follow-up FGDs were not possible due to school closures during the COVID-19 pandemic. This limited our ability to revisit emerging themes with participants. While FGDs are less suited than individual interviews for probing deeply into traumatic personal experiences. However, they provided an important lens into the shared meanings, peer dynamics, and social processes through which SRGBV is perceived and enacted in schools. Additionally, the distribution of quotations across participants was uneven. A few girls provided especially detailed accounts of SRGBV, while boys’ voices appeared less frequently and mainly in relation to bystander experiences. This imbalance likely reflects the gendered distribution of direct SRGBV exposure, but it limits the breadth of male perspectives represented. Nonetheless, the two FGDs generated meaningful insights.

Manual coding of the data, while encouraging close reading, was time-consuming and may introduce inconsistencies. To reduce this risk, the coding was done by more than one researcher, with regular discussions to agree on the themes. The small number of participants means the findings cannot be generalized. However, the study offers a clear picture of how SRGBV is experienced in one school and raises questions that deserve further investigation. The small number of participants means the findings cannot be generalized. We also acknowledge that with only two FGDs theoretical saturation was not achievable. Rather than definitive conclusions, the findings should be viewed as exploratory, hypothesis-generating insights into SRGBV in one school context. Nevertheless, they offer a clear picture of how SRGBV was experienced and raise questions that deserve further investigation. Future research should involve more schools, repeat data collection over time, and focus directly on SRGBV to build on these findings.

## Conclusion

This study underscores the embedded nature of SRGBV within the everyday experiences of students in one co-educational secondary school. These accounts are context-specific, though they raise questions that may resonate in broader settings. Through thematic analysis of participant narratives, patterns emerged that highlight how symbolic violence provides a useful framework for interpreting the normalization and persistence of SRGBV. Importantly, this form of violence does not operate in isolation. Rather, it is intertwined with structural constraints and cultural norms that appear to legitimize and sustain harmful behaviors within the school environment. The analysis suggests that the absence of confidential reporting mechanisms, alongside prevailing attitudes that minimize harassment and shift blame onto survivors, creates conditions under which violence is reproduced. These findings point to the need for an integrated response that goes beyond reactive or punitive measures. Effective interventions must also confront the deeper social norms and institutional arrangements that render SRGBV both visible and invisible in daily school life.

Recommendations drawn from the thematic insights include the establishment of survivor-centered and anonymous reporting pathways, the provision of trained counselors for follow-up support, and gender-responsive training for teachers to foster accountability and empathy. Peer-led initiatives may be promising avenues for disrupting complicity and promoting collective responsibility among students. Furthermore, the themes indicate the importance of engaging families and broader community actors to address the intergenerational transmission of gendered norms that shape school environments. From a public health perspective, these findings highlight schools as key entry points for early detection of psychosocial distress linked to SRGBV. Strengthening teacher training, embedding psychosocial support, and integrating gender-sensitive policies are critical to reducing downstream risks of depression, substance use, and suicidality.

Finally, the findings highlight the psychosocial burden of SRGBV, as interpreted through students’ accounts of mistrust and reluctance to seek institutional support. These concerns point to the value of embedding trauma-informed mental health services within school structures. Taken together, the conclusions drawn from participants’ experiences suggest that a holistic approach, combining structural reform, cultural transformation, and psychological care, is essential for fostering safer and more equitable school spaces.

## Supplementary Information

Supplementary Material 1.

## Data Availability

Data provided in the supplementary files.

## Abbreviations

EDCTP2: European & Developing Countries Clinical Trials Partnership Programm
FGD: Focus Group Discussion
GBV: Gender-Based Violence
K-RITH: KwaZulu-Natal Research Institute for Tuberculosis and HIV
PTSD: Post-Traumatic Stress Disorder
SANTHE: Sub-Saharan African Network for TB/HIV Research Excellence
SRGBV: School-Related Gender-Based Violence
UNESCO: United Nations Educational, Scientific and Cultural Organization
UNGEI: United Nations Girls’ Education Initiative
