# Supplementary material for: A descriptive phenomenological study of school-related gender-based violence: lived experiences of symbolic violence, harassment, and systemic complicity in a mixed secondary school in Nairobi, Kenya
Source: BMC Public Health. 2025 Nov 12;25:3926. doi: 10.1186/s12889-025-25341-0 (PMC12613922; doi:10.1186/s12889-025-25341-0)
Supplement: Supplementary file 1 — Supplementary Material 1. [file 12889_2025_25341_MOESM1_ESM.docx]

School Related Gender Based Violence Experiences and Themes

| **Quote** | **Coding** | **Labels** | **Subthemes** | **Themes** | **Psychological Influence** | **Societal Implications** |
| --- | --- | --- | --- | --- | --- | --- |
| When going for lunch, those big boys from Form Three and Form Four come and push you back, and you can’t do anything... (Naliaka, female, 14-15 years) | Physical dominance, exclusion | Bullying, power imbalance, helplessness | Hierarchical hegemony | **Power Assertion and Hierarchical Violence** | Helplessness and fear | Normalization of hierarchical power relations |
| Sometimes you are walking down the stairs, and then a boy kicks you. When you tell him, 'I will report to the teacher that you caused me to fall,' he tells you, 'Try doing that, and I will slap you.' (Naserian, female, 16-17 years) Unaona lunch time, they… wanakutumia. Sasa tuseme hakuna maprefects wengi pahali, wanachukua chakula yako, wanagawana. Unabakishiwa kama two spoons, na unaambiwa ukijaribu kusema, utaona. (You see, at lunchtime they …use you. If there aren’t many prefects around, they take your food and share it among themselves. You’re left with just two spoonfuls and warned that if you say anything, there’ll be consequences).(Kiprotich, male, 14-15 years) | Fear enforcement, retaliation | Physical assault, retaliation, silencing | Fear-driven silencing and retaliation |  | Anxiety and fear of retaliation | Institutional failure to protect victims |
| They will wait for you outside the school, or they start planning and getting information. You start fearing even in school or wanakupiga (they beat you up). (Naserian, female, 16-17 years) | Organized violence, premeditation | Surveillance, fear, premeditated violence | Strategic use of fear and intimidation |  | Constant fear and hyper-vigilance | Schools as unsafe spaces |
| Say someone in the field hits you with a ball he says wee mtoto nyamaza (Shut up, you child). (Odhiambo, male, 16-17 years) | Infantilization, verbal control | Humiliation, verbal silencing | Infantilization and verbal domination |  | Emotional suppression | Reinforcement of hierarchical control through dismissal |
| Wengine wakikupata kwa line wanakuita form one ama wanakuita na admission yako kuja unichote maji. Imagine, anakuita na admission. (Wanjiku, female, 16-17 years) (Some others, when they get you lining up to fetch water, they call you Form One or by your admission number and tell you, “Come and draw water for me.”) | Dehumanization, verbal abuse | Exclusion, verbal abuse | Dehumanization and power assertion |  | Feeling of inferiority and exclusion | Maintenance of hierarchical social control |
| Sometimes you are walking within the school, and boys in Form 3 and 4 will corner you somewhere and want to touch you, to touch your breasts or your buttocks. If you refuse, they start saying, 'Ona huyu sura mbaya' (Look at this ugly one). It makes me feel bad and sometimes I just sit in class and think why God let my parents bring me to this school. (Naliaka, female, 14-15 years) | Forced physical contact, peer shaming | Sexual coercion, peer judgment, humiliation | Coercive social expectations | **Sexual Harassment and Normalization of Coercion** | Emotional distress and loss of self-worth | Social acceptance of gendered harassment |
| Na some of these guys… tuseme hii shule kuna wale ambao wana abuse drugs. Unajua saa ukiabuse drugs, mental yako venye unakaa. Kuna ninii apo, challenges, so unajua mtu kama huyo haskii vibaya na haoni kitu kubwa—yaani big deal—kushika msichana. Unajua wasichana ndo wakona iyo shida. Kijana unaeza manage kama sisi vijana wadogo. Huyu msichana ni msichana, hana nguvu, yaani tu sijui nisemeaje. (Some of these guys… let’s say in this school there are some who abuse drugs. And you know when someone abuses drugs, their mental state changes. They face certain challenges, so someone like that doesn’t feel bad or even see it as a big deal to touch a girl. You see, it is the girls who really face that problem. A boy might be able to handle it, like we smaller boys. But a girl is a girl, she is powerless, I don’t even know how to explain it.) (Mwangi, male, 14-15 years) | Substance use, justification of harassment, gendered vulnerability, normalization | Impaired empathy, excusing violence, perceived female helplessness | Substance-enabled minimization of violence |  | heightened vulnerability due to drug-fueled aggression | Substance use as a pre-text for gender based violence |
| Like the other day, I was leaving school, I took my bag, and was heading downstairs when a boy came up to me and said, 'Why don’t you give me a hug?' I told him, 'I don't like that behavior.' Then he started saying, 'Oh, a hug is like a greeting for us.' (Naserian, female, 16-17 years) | Coercion, dismissal of boundaries | Pressure, unwanted physical contact | Normalization of gendered coercion |  | Erosion of personal boundaries | Societal trivialization of consent |
| Nilijaribu kuambia my mum that hawa maboys wamekuwa wakinishikashika. Alinislap na akaniambia 'Si nilikuambia uwache hii umalaya uko nayo!' (Kerubo, female, 14-15 years) (I tried telling my mum that these boys were touching me. She slapped me and said, “Didn’t I tell you to stop being promiscuous?”) | Victim-blaming, parental rejection | Family-based silencing, moral policing | Custodial victim blaming | **Systemic Complicity and Suppression** | Self-blame and isolation | Reinforcement of harmful gender norms |
| The teacher told me to dress properly if I didn’t want attention. (Kerubo, female, 14-15 years) | Victim-blaming, institutional neglect | Victim-blaming by teachers, moral policing |  |  | Guilt and emotional withdrawal |  |
| There are students who, when they see you talking to teachers, think you are 'snitches.' But teachers also can’t be trusted for they tell other teachers issues to do with students and this makes some students go mute on reporting bullying in the school. (Baraka, male, 16-17 years) | Institutional betrayal, mistrust | Distrust of authority, silencing mechanisms | Victim silencing |  | Suppression of grievances | Weak trust in institutional structures |
| If you are appointed as a prefect and you write their names down as noisemakers and take them to the teacher, they will wait for you outside the school [to deal with you]. (Naserian, female, 16-17 years); “Yes…you have to go with your sister or brother or someone who is older than you to protect [you] from those people. Let’s say we went to a CU [Christian Union meeting] and talked about something that hurt them something that they do they want to beat you up. Me what I do I look for somebody mwenye tunaishi nayeye (who is my neighbour) then we go home because I fear those girls” (Achieng, female, 14-15 years) | Retaliation; threat of violence | Fear of leadership roles, societal risk beyond administrative sphere of control | External suppression |  | Fear and avoidance of leadership roles | Weak enforcement of school rules |
| Some of the older girls just accept to be touched and they laugh at us and say, 'Nyinyi ni washamba, kuja tuwafunze' (You are backward, come, let us teach you). I ran away and hid myself in the toilet and I was thinking about what they were saying. Hadi unapata msichana anakalia juu ya kijana hadi unafikiria…(You even find a girl sitting on a boy and you are left thinking…) (Naliaka, female, 14-15 years)  Eeh (Yes)… a boy anakaa hivi apart and then msichana anakalia (A boy sits like this with his legs apart and then the girl sits in between) . (Naserian, female, 16-17 years) | Peer complicity, internalized patriarchal norms | Gatekeeping of harassment, peer reinforcement | Harassment gatekeeping | **Reinforcement of Violence** | Shame, emotional conflict | Reinforcement of social policing through peer expectations |
| Or anatuma watu wenye anajua wanakuanga wabaya... (He sends his dangerous associates who waylay you.) He won’t be there so you won’t know it’s him. Then ukimsuspect (if you suspect him) he starts saying he wasn’t uiliniona (Did you see me there)? I wasn’t there (Wanjiku, female, 16-17 years) | Threats through proxies, covert control | Indirect intimidation, fear | Covert power |  | Fear and disempowerment | Maintenance of covert power structures |
| I ran away and hid myself in the toilet and I was thinking about what they were saying. (Naliaka, female, 14-15 years) | Avoidance, emotional withdrawal | Coping through isolation | Avoidance and emotional withdrawal, internalized blame | **Psychosocial Impact and Internalized Harm** | Fear and isolation | Long-term emotional and social withdrawal |
| I don’t talk to teachers anymore because they don’t help. (Baraka, male, 16-17 years)  “Uhmmm…. Some teachers ignore you when you report bullying to them. They tell you it’s not a big thing just deal with it as a man since some of these challenges you will face anywhere” (Odhiambo, male, 16-17 years)  “…There are students who, when they see you talking to teachers, think you are 'snitches.' But teachers also can’t be trusted for they tell other teachers issues to do with students and this makes some students go mute on reporting bullying in the school.” (Baraka, male, 16-17 years) | Mistrust of authority, emotional withdrawal | Long-term avoidance of authority figures | Distrust in custodial authority |  | Mistrust and withdrawal | Erosion of trust in education systems |

*Note. These are not real names but pseudonyms*
